# Supplementary material for: A new megaspilid wasp from Eocene Baltic amber (Hymenoptera: Ceraphronoidea), with notes on two non-ceraphronoid families: Radiophronidae and Stigmaphronidae
Source: PeerJ. 2018 Aug 8;6:e5174. doi: 10.7717/peerj.5174 (PMC6103384; doi:10.7717/peerj.5174)
Supplement: Table S1 [file peerj-06-5174-s001.pdf]

	DEI-GISHym31820 (paratype, 1013)	DEI-GISHym31819 (holotype, 1805)
**Body Length**	2411.09 µm	2007.29 µm
**HW**	676.87 µm	541.52 µm
**HH**	572.05 µm	454.28 µm
**OOL**	111.15 µm	90 µm
**POL**	119.95 µm	465 µm
**LOL**	103.55 µm	127.41µm
**Ped L**	133.69µm	106.75 µm
**Sc**	503.925µm	452.865 µm
**F1**	145.105µm	122.515 µm
**F2**	92.995µm	78.84 µm
**F6**	104.055µm	83.145 µm
**F7+F8 length**	175.775µm	173.985 µm
**HW**	676.87 µm	541.52 µm
**IOS**	363.70 µm	269.27 µm
