# Supplementary figures and images for: A new megaspilid wasp from Eocene Baltic amber (Hymenoptera: Ceraphronoidea), with notes on two non-ceraphronoid families: Radiophronidae and Stigmaphronidae
Source: PeerJ. 2018 Aug 8;6:e5174. doi: 10.7717/peerj.5174 (PMC6103384; doi:10.7717/peerj.5174)

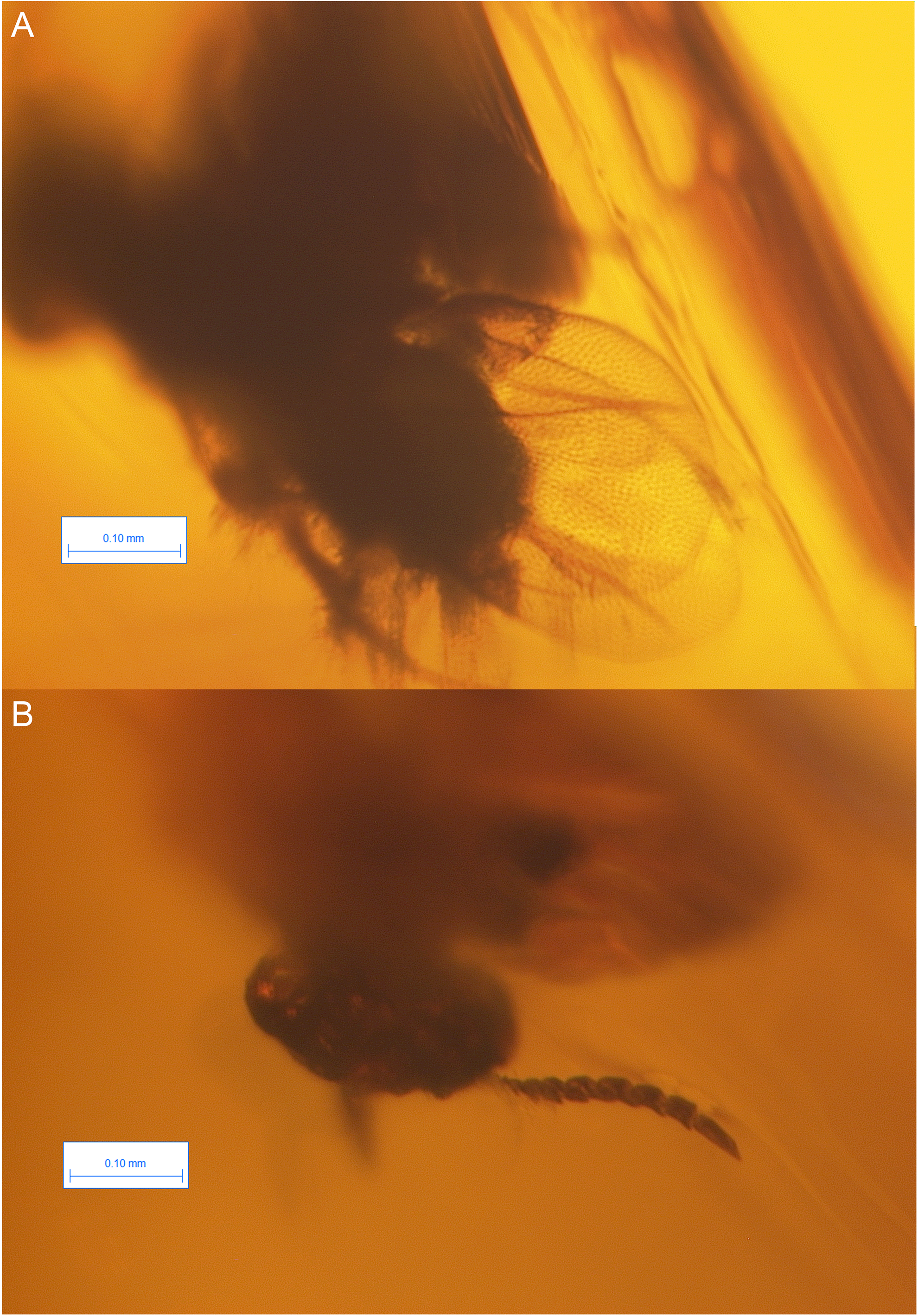

Supplement: Figure S2 — This species is considered now as incertae sedis. [file peerj-06-5174-s003.png]

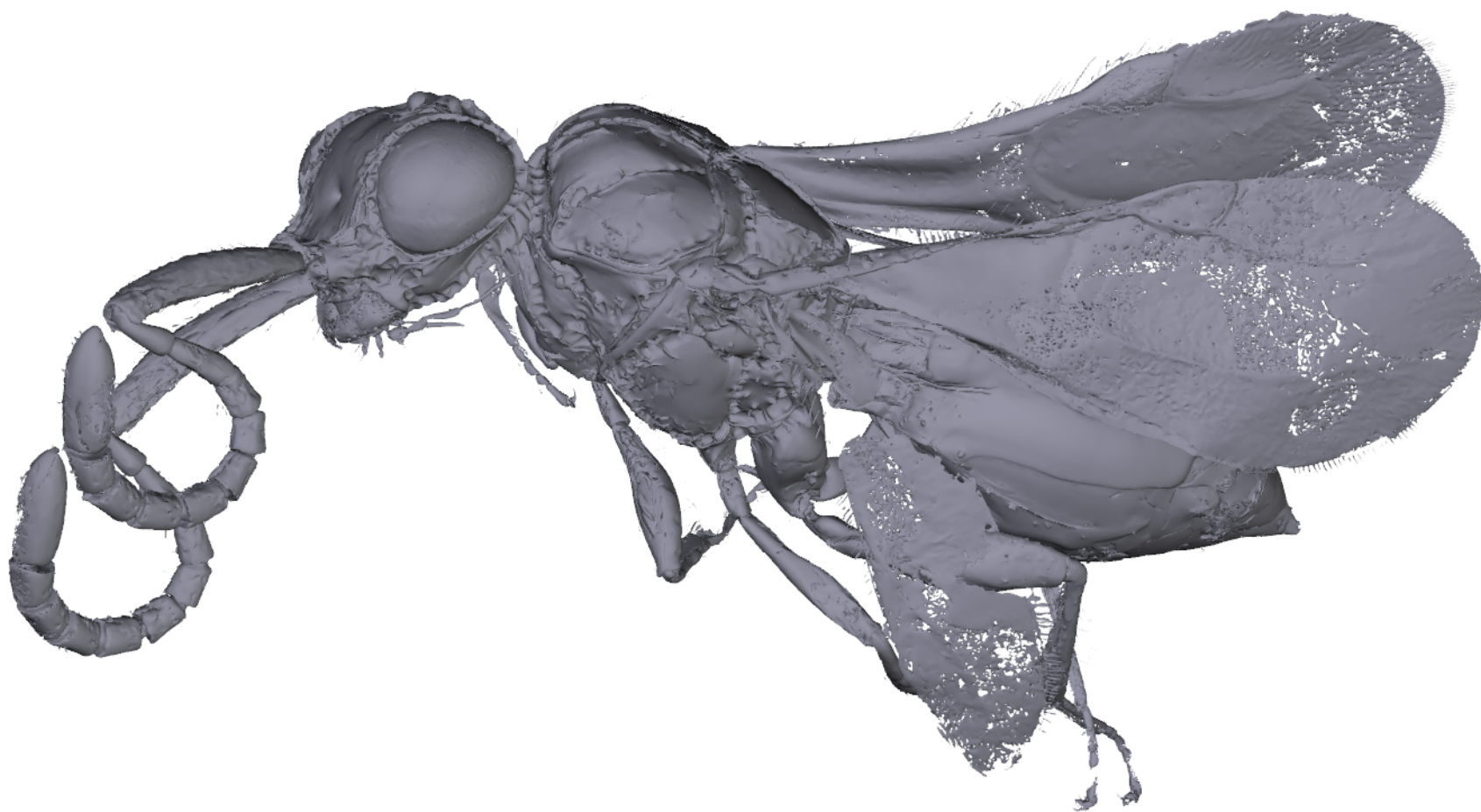

Supplement: File S1 [file peerj-06-5174-s004.pdf]
